# Comparison of maternal serum lipoproteins in normal pregnancy and primiparous patients with eclampsia

**DOI:** 10.12669/pjms.324.9859

**Published:** 2016

**Authors:** Rubina Nazli, Tasleem Akhtar, Nabila Sher, Jamila Haider, M. Akmal Khan, Hina Aslam

**Affiliations:** 1 Dr. Rubina Nazli, MBBS, PhD. Assoc. Prof. of Biochemistry, Institute of Basic Medical Sciences, Khyber Medical University, Peshawar, Pakistan; 2 Dr. Tasleem Akhtar, M. Phil, PhD Chemistry. Senior Scientific Officer (Rtd), PMRC Research Centre, Khyber Medical College, Peshawar, Pakistan; 3 Dr. Nabila Sher Mohammad MBBS, M.Phil. Assistant Professor of Biochemistry, Khyber Girls Medical College, Peshawar, Pakistan; 4 Jamila Haider, BS Microbiology, Dept. of Microbiology, Swat University, KPK, Pakistan; 5 Dr. Mohammad Akmal Khan, MBBS, MCPS, FCPS. Psychiatry Unit, Hayatabad Medical Complex, Peshawar, Pakistan; 6 Dr. Hina Aslam, MBBS, FCPS-1. Gynea Unit, Hayatabad Medical Complex, Peshawar, Pakistan

**Keywords:** Primiparous women, Hypertension, Eclampsia, Lipoproteins

## Abstract

**Objective:**

To evaluate changes in serum lipoproteins in primiparous women with eclampsia and compare it with pregnant women having normal blood pressure.

**Methods:**

This cross sectional study was conducted on 65 primiparous eclamptic patients and 21 normotensive pregnant women in the tertiary care hospitals of Peshawar. History of each woman was recorded on a questionnaire. Blood pressure was measured using standard methods. About 5 ml of venous blood was drawn for the analysis of lipoproteins. The data was analyzed using computer software package SPSS version 10. The P value <0.05 was considered statistically significant.

**Results:**

Mean age of hypertensive cases was 23.2 ± 0.52 years while that for controls was 23.9 ± 1.16 years. Significant differences were found in serum lipoproteins. Women having eclampsia had 28.8%, 29.5%, 31.1%, 32.9% and 65.3% higher, low density lipoprotein-cholesterol (LDLC), triglycerides (TG), total cholesterol (TC):high density lipoprotein-cholesterol (HDLC) ratio, LDLC: HDLC ratio and TG: HDLC ratio respectively as compared to the control group. The HDLC concentrations, HDLC: VLDLC ratio and apolipoprotein-A1 level were 26.9%, 56.6% and 27.9% respectively, lower in the patient group as compared to the controls.

**Conclusion:**

This study suggests that evaluation of lipoprotein concentrations during antenatal period can be helpful in the early detection and prevention of developing eclampsia.

## INTRODUCTION

Eclampsia is a complex multisystem hypertensive disorder seen worldwide during late pregnancy and is a leading cause of maternal and fetal morbidity and mortality.^1^ It is a dangerous complication of pregnancy with a sudden onset and has the features of developing tonic – colonic seizures in a patient who has pre-eclampsia. The risk of developing pregnancy induced hypertension (PIH) increases with increasing age.^2^ It is usually associated with placental hypoxia and dysfunction.^3^

It is characterized by blood pressure of 140/90 mm of Hg or rise in systolic blood pressure of more than 30mm of Hg or diastolic blood pressure of more than 15mm of Hg after 20 weeks of gestation accompanied by proteinurea ≥ 300mg / 24 hrs or greater or equal to 1+ or 100mg /dl by dipstick response.^4^,^5^

Pre-eclampsia/eclampsia occurs during second and third trimester of pregnancy and is a more common problem usually associated with a woman’s first pregnancy in nulliparous women (primigravida). Nearly 7 to 10% of pregnancies are complicated with PIH in developing countries.^6^ In India, the national incidence of pregnancy induced hypertension is 15.2%, with the incidence in nulliparous women being four times greater than in multipara.^7^ In Berhampur, Orissa, India maternal deaths due to PIH was 32% which is twice the national incidence of India.^8^,^9^

In Nigeria the incidence is higher in the Northern part of the country with prevalence rate of 17% and accounts for up to 40 percent of maternal deaths. Given the tendency toward and culture of early marriage in the North Nigeria, the majority of those affected by this condition are teenagers. (Population Council FMOH 2009).

In Pakistan^10^ the prevalence of pre-eclampsia and eclampsia is reported around 19%. A study from Hayatabad Medical Complex^11^, Peshawar-Pakistan reported maternal mortality from eclampsia as 16.7% that accounted for 29.4% of the total maternal deaths during one year period.

Changes in lipoproteins in essential hypertension are documented.^12^ Serum lipids increase significantly during pregnancy and are further elevated two fold during PIH.^13^ Abnormal lipoprotein levels are responsible for damage to the endothelium which leads to high blood pressure and proteinuria; which are important signs of PIH.^14^

Change in lipoproteins cause atherosclerosis, damage to endothelium and other heart diseases. The major sign of PIH is hypertension, suggesting that it is due to vasospastic events in placenta, uterus and brain.^15^

The present study has been undertaken to compare the changes in maternal serum lipoprotein in normal pregnancy and primiparous patients with eclampsia.

## METHODS

This cross sectional study was conducted in the pregnant women admitted in the obstetrics and gynecology department of tertiary care hospitals of Peshawar in the Khyber Pakhtunkhwa Province of Pakistan. A total of 86 pregnant women comprising 21 normotensive primiparous women and 65 primiparous women with eclampsia at gestational age of >20 weeks were registered in the study after taking informed consent. The study was approved by the Institutional Research and Ethical Board of the Postgraduate Medical Institute, Hayatabad Medical Complex, Peshawar.

### Exclusion criteria

The cases and controls having past history of diabetes mellitus, hypertension, renal disease, liver disorders, multiple pregnancies, those with family history of eclampsia and history of treatment with drug influencing lipid profile were excluded.

### Inclusion criteria

Cases of eclampsia primi patients with gestation age more than 20 weeks. The eclamptic patients were diagnosed by the presence of persistent hypertension (140/90 mm of Hg or more), gross proteinurea (tested by heat test of urine) with or without oedema. History of patients was taken and blood pressure of each subject was recorded using standard methods.

About 5 ml of venous blood sample was obtained from both cases and controls in a gel vaccutainer. All specimens were transferred over ice cubes to PMRC Research Centre, Khyber Medical College Peshawar for further investigations. Serum triglycerides and total cholesterol were measured by enzymatic method of Elitech diagnostic kits of France.

Serum high density lipoprotein cholesterol was measured by using kits of Merck Diagnostics (Germany). Serum very low density lipoprotein cholesterol was calculated as 1/5 of triglycerides. Serum low density lipoprotein cholesterol was calculated by Frederickson-Friedwald’s formula according to which LDL-C = TC-HDL-C-VLDL cholesterol. Serum lipids were analyzed by using semi auto chemistry analyzer Microlab 200 (Merck). Serum apolipoprotein A1 (APO-A1), apolipoprotein B100 (APO-B100) and lipoprotein-(a) (LPA) were measured in the Pathology Laboratories of Postgraduate Lady Reading Hospital, Peshawar using turbiditrimetric kits of Roche Diagnostics, on chemistry auto analyzer, modular P-800 by Roche, Cobas (Japan).

Student’s *t*-test was used for statistical significance. A level of P <0.05 was accepted as statistically significant. SPSS program version 10.0 was used for statistical analyses (SPSS Inc, Chicago, Illinois, USA). The results are presented as mean values ± SEM (Standard Error of Means).

## RESULTS

Mean age and gestational age of the patient group was comparable with the control group, while systolic and diastolic blood pressure of the patient group was considerably raised and was highly significant when compared with the control group (Table-I).

**Table-I:** Comparison of different variables of primiparous pregnant women with the control group.

Variables	Patients Mean± SEM	Control Mean± SEM	P
Age (years)	23.15± 0.52	23.90± 1.16	NS
Gestation age (weeks)	31.64 ± 0.58	30.81± 1.03	NS
Systolic BP (mmHg)	158.79 ± 2.82	115.23± 2.05	0.001
Diastolic BP (mmHg)	105.59 ± 2.02	76.04± 1.54	0.001

Mean ± SEM of blood lipid levels in normal pregnancy and in primiparous women with eclampsia are shown in Table-II. Significant differences were found in serum high density lipoprotein-cholesterol (P <0.001), very low density lipoprotein-cholesterol (P <0.001), triglycerides (P <0.001), total cholesterol:high density lipoprotein-cholesterol ratio (P <0.001), serum triglycerides:high density lipoprotein-cholesterol (P <0.001), high density lipoprotein-cholesterol:very low density lipoprotein-cholesterol ratio (P <0.01) and apolipoprotein A1 level (P <0.001) among the groups. However, no significant difference (P >0.05) was noted in the total cholesterol, low density lipoprotein-cholesterol, apolipoprotein B100, low density lipoprotein:apolipoprotein B100 ratio and high density lipoprotein:apolipoprotein A1 in eclamptic patients and normotensive pregnant women.

**Table-II:** Comparison of lipoprotein concentrations in primiparous women with eclampsia and normal pregnancy.

Parameters	Eclampsia group Mean ± SEM	Control group Mean ± SEM	% deviation from control	P
Total Chol. (mg/dL)	213.61 ± 6.84	206.28 ±16.47	3.6 ⇑	NS
HDL-Chol. (mg/dL)	40.72 ± 1.16	53.20 ± 2.36	23.5 ⇓	0.001
LDL-Chol. (mg/dL)	109.55 ± 6.37	106.55 ± 14.37	2.8 ⇑	NS
VLDL-Chol. (mg/dL)	65.12 ± 3.51	44.7 ± 3.57	45.7 ⇑	0.001
Triglyceride (mg/dL)	327.98 ± 17.78	223.4 ± 17.87	46.8 ⇑	0.001
APO-A1 (mg/dL)	143.59 ± 5.85	189 ± 11.31	24.0 ⇓	0.001
APO-B100 (mg/dL)	118.98 ± 4.96	126.18 ± 3.25	5.70 ⇓	NS
TC:HDL-C ratio	5.38 ± 0.19	3.95 ± 0.36	36.2 ⇓	0.001
LDL-C:HDL-C ratio	2.76 ± 0.16	2.07 ± 0.29	33.3 ⇑	0.01
TG:HDL-C ratio	8.38 ± 0.50	4.41 ± 0.43	90.0 ⇑	0.001
HDL-C: VLDL-C ratio	0.72 ± 0.03	1.30 ± 0.09	44.6 ⇓	0.001
LDL: APO-B100	0.99 ± 0.06	0.95 ± 0.14	4.2 ⇑	NS
HDL: APO-A1	0.31 ± 0.02	0.28 ± 0.03	10.7 ⇑	NS

Women having eclampsia had 28.8%, 29.5%, 31.1%, 32.9% and 65.3% higher, low density lipoprotein-cholesterol, triglycerides, total cholesterol:high density lipoprotein-cholesterol ratio, low density lipoprotein-cholesterol:high density lipoprotein-cholesterol ratio and triglycerides:high density lipoprotein-cholesterol ratio respectively as compared to the control group. The high density lipoprotein-cholesterol concentrations, high density lipoprotein-cholesterol:very low density lipoprotein-cholesterol ratio and apolipoprotein-A1 were 26.9%, 56.6% and 27.9% respectively, lower in the patient group as compared to the controls.

## DISCUSSION

Elevation of serum lipids during pregnancy and in pregnancy induced hypertension have been reported by several investigators.^16^,^17^ Remarkably high changes have been noted in the triglycerides, which may go as high as two to three folds in the third trimester of pregnancy.^18^ The same pattern was seen in the present study where a significant rise in serum triglycerides was noted in eclampsia patients when compared to women with normal pregnancy.

The hyperoestrogenemic state of pregnancy is mainly responsible for this rise in serum triglyceride level in pregnancy. Oestrogen induces hepatic bio-synthesis of endogenous triglyceride which is carried by VLDL.^19^

Increased triglycerides, found in PIH^20^ is likely to be deposited in predisposed vessels, such as the uterine spiral arteries and contribute to the endothelial dysfunction, both directly and indirectly through generation of small, dense LDL.

Mohanty et al.^21^ have found significant increase in serum cholesterol in toxemia of pregnancy in primiparous patients. Our results are contrary to these findings where cholesterol concentration increased upto certain level but no significant alteration in total cholesterol level was observed. These results are consistent with the findings reported in other studies.^20^,^22^ However in the present study, the mean HDL-C was about 24% lower in the eclampsia patients than in pregnant women with normal pregnancy (P<0.001). Other investigators have also reported similar results.^23^,^24^

In present study, the change in LDL-C cholesterol was not significant in patients and controls, however serum VLDL-C levels rose significantly (P<0.001) in the eclampsia group, which may be due to hyper-triglyceridemia leading to enhanced entry of VLDL that carries endogenous triglycerides into circulation. The VLDL-C level, as reported by others^25^ might raise to 2.5 folds at term over the pre-pregnancy level. VLDL levels further increase in eclampsia as found in the present study and also reported by others.^20^,^26^

In the present study, levels of APO-B100 concentrations decreased in the patients group but not up to a significant value. However, a significant decrease in APO-A1 levels was observed in the patient group that are likely to originate from polymorphism of APO-A1 of HDL and/or functional disorder of HDL.

The ratios between different lipids like LDL-C: HDL-C; TC: HDL-C and TG: HDL-C were calculated. There was a significant increase in LDL-C: HDL-C, TC: HDL-C and TG: HDL-C ratio while a significant decrease was noted in HDL-C: VLDL-C ratio in patient group as compared to normotensive pregnant women. These results are comparable with another study reported by Enquobahrie et al.^27^ Although the importance of these ratios in pregnancy and eclampsia is not yet recognized, the significance of altered lipid ratios cannot be ignored as they point to additional risks in eclampsia patients.
